# Six-month outcomes of the CODES randomised controlled trial of cognitive behavioural therapy for dissociative seizures: A secondary analysis

**DOI:** 10.1016/j.seizure.2022.01.016

**Published:** 2022-03

**Authors:** Laura H. Goldstein, Emily J. Robinson, Trudie Chalder, Markus Reuber, Nick Medford, Jon Stone, Alan Carson, Michele Moore, Sabine Landau

**Affiliations:** a Department of Psychology, King's College London, Institute of Psychiatry, Psychology and Neuroscience, DeCrespigny Park, London SE5 8AF, UK; b School of Population Health and Environmental Sciences, King's College London, UK; c Research Data and Statistics Unit, Royal Marsden Clinical Trials Unit, The Royal Marsden NHS Foundation Trust, Surrey, UK; d Department of Psychological Medicine, King's College London, Institute of Psychiatry, Psychology and Neuroscience, UK; e Academic Neurology Unit, Royal Hallamshire Hospital, University of Sheffield, Sheffield, UK; f South London and Maudsley NHS Foundation Trust, London, UK; g Centre for Clinical Brain Sciences, University of Edinburgh, UK; h Centre for Social Justice and Global Responsibility, School of Law and Social Sciences, London South Bank University, London, UK; i Department of Biostatistics and Health Informatics, King's College London, Institute of Psychiatry, Psychology and Neuroscience, UK

**Keywords:** Dissociative seizures, Cognitive behavioural therapy, Randomised controlled trial, Outcomes

## Abstract

**Purpose:**

The CODES Trial for adults with dissociative seizures had a predesignated 12-month post-randomisation follow-up point for outcome evaluation. We undertook an exploratory, unplanned, secondary analysis to evaluate the effectiveness of cognitive behavioural therapy plus standardised medical care (CBT+SMC) compared to SMC alone at 6 months post-randomisation, i.e., closer to the end of treatment.

**Methods:**

The analysis of 6-month data followed our previous method of using multiple imputation and an intention-to-treat approach to analyse variables 12 months post-randomisation.

**Results:**

The original trial primary outcome of monthly seizure frequency showed greater benefit from CBT+SMC than SMC-alone at 6 months (at *p* < 0.05). Of 13 comparable previously-defined secondary outcomes, 12 showed a significant between group effect (*p* < 0.05) in favour of the CBT intervention at 6 months. The average effect size of the comparable previously-defined primary and secondary continuous outcomes was 0.33 at 6 months vs 0.26 at 12 months. The estimated Incidence Rate Ratio (IRR) quantifying monthly seizure reduction was IRR = 0.72 (95%CI from 0.55 to 0.93) at 6 months compared to IRR = 0.78 at 12 months.

**Conclusion:**

DS-specific CBT (plus SMC) produced evidence of significant benefits at 6 months post- randomisation (around which time CBT was complete) compared to SMC alone; for the majority of these outcomes, better results following CBT (plus SMC) had previously been reported at 12 months. Our pattern of results suggests that short- and longer-term follow-ups are necessary to understand treatment effects in this disorder. Studies only providing short-term follow-up data should be interpreted with caution.

## Highlights

• We report an unplanned secondary analysis of 6-month outcomes in the CODES trial.
• 13/14 outcomes were better following CBT plus standardised medical care (SMC).
• Monthly seizure frequency was significantly lower at 6 months in the CBT+SMC group.
• Treatment effect sizes across outcomes were moderate-to-large at 6-month follow-ups.
• Short- and longer-term evaluations are necessary to understand treatment effectiveness.

## 1 Introduction

While psychological interventions are considered to be the treatment of choice for adults with dissociative seizures (DS), evidence for such treatments was limited prior to the ‘Cognitive behavioural therapy vs standardised medical care for adults with Dissociative non-Epileptic Seizures (CODES)’ Trial. The CODES trial set out to investigate the effectiveness of standardised medical care (SMC) alone versus DS-specific Cognitive Behavioural Therapy (CBT) plus SMC for adults with DS [1], [2], [3]. Before completion of the CODES trial only two pilot randomised controlled trials (RCTs) had been completed using different CBT approaches [4,5] which suggested efficacy for CBT-based interventions. In Goldstein et al.’s [4] study, which randomised 66 patients, there was evidence of significant benefit from a DS-specific CBT intervention at the end of treatment compared to neuropsychiatric care (p=0.002; between-groups effect size = 0.75) although, at a 6-month follow-up post end of treatment, the difference between the groups was no longer significant (*p* = 0.082; between-groups effect size = 0.42). LaFrance et al. [5] evaluated data from a total of 34 of 38 recruited patients randomised across four treatment arms at week 16. Their study was not powered to allow between-group comparisons and only within-arm comparisons were made, which are not directly comparable to the data in the study above. LaFrance et al. [5] found that CBT-informed psychotherapy, alone or with sertraline, led to a reduction in DS frequency (Slope [95%CI]: -0.72 [-1.3, -0.2] *n* = 9; and -0.90 [-1.6, -0.2] *n* = 9, respectively). Improvements in secondary outcomes included global functioning.

The CODES trial was a fully-powered, parallel arm multi-centre RCT designed to address the limitations in the evidence for psychotherapeutic interventions for people with DS. Results indicated that at 12 months, which was the predesignated endpoint, there was no significant difference between groups in the primary outcome (monthly DS frequency) [2], [3]. This broadly corresponded to the final timepoint in the pilot RCT [4]. Differences between groups were also significant in several important secondary outcomes in favour of CBT+SMC [2], [3]. At 12 months post-randomisation, the CBT+SMC group rated their DS as less bothersome [6] (*p* = 0.020), they reported a longer number of consecutive days without DS in the previous six months (*p* = 0.001), better functioning as measured on the Work and Social Adjustment Scale [7] (*p* < 0.001), fewer somatic symptoms on a modified PHQ-15 [8] (*p* = 0.008), less distress as measured on the CORE-10 [9] (*p* = 0.013) and better health ratings on the visual analogue scale from the EQ-5D-5L [10] (*p* = 0.010). In addition, the CBT+SMC group had better self-rated (*p* = 0.001) and clinician-rated (*p* < 0.001) global outcomes and were more satisfied with the treatment they had received (*p* < 0.001). At the 12-month follow-up we did not demonstrate significant differences in the proportions of people showing a >50% reduction in seizures or in the proportions of people who were seizure free in the last three months of the study. Neither did the two groups differ significantly in their ratings of seizure severity, anxiety, depression or on the Mental or Physical Component Score on the SF-12v2 [11]. Where significant differences were found these reflected moderate effect sizes. At 12 months post-randomisation none of the secondary outcome measures favoured the SMC-alone group.

However, to further inform clinical practice, to understand better the pattern of responses to our interventions and to determine whether there was a treatment effect in favour of our CBT+SMC group close to the end of the treatment delivery (i.e., at 6 months post-randomisation), it is important to understand which outcomes may have been better in the CBT+SMC intervention arm compared to SMC-alone at the 6-month post-randomisation timepoint. An exploration of the pattern of change in the two arms of the study may provide information about the impact of the different aspects of treatment provided. It may also help with the development and optimal management pathways in the future. In addition, this analysis may inform the understanding of the results of other studies with shorter follow-ups, and where the sample sizes were considerably smaller than in the CODES study. In a recent systematic review of different psychotherapies for DS [12], seven studies of varying designs [5,[13], [14], [15], [16], [17], [18]] chose to evaluate outcomes only at the end of treatment. All studies were small and likely underpowered. The median number of participants was 37 (range 6–60). In this paper, we report an exploratory, unplanned, secondary analysis of the fully powered CODES trial data (*n* = 368) to evaluate the effectiveness of the CBT+SMC intervention, compared to SMC alone at 6 months post-randomisation. This may shed light on the extent to which CBT is effective for people with DS, close to treatment end and in a substantially larger sample than in previous studies.

## 2 Methods

### 2.1 Description of CODES Trial CBT+SMC and SMC alone

The CODES trial was a pragmatic, parallel-arm, multi-centre randomised controlled trial that randomised 368 people with DS to receive either SMC alone (*n* = 182) or DS-specific CBT (12 sessions plus a booster session) plus SMC (CBT+SMC *n* = 186). Following initial recruitment of 698 people from 27 neurology or specialist epilepsy services in England, Wales and Scotland into a screening phase between October 2014 and February 2017, 368 patients were randomised (from 17 liaison psychiatry / neuropsychiatry services) using a 1:1 ratio to SMC or CBT+SMC, stratifying by liaison psychiatry / neuropsychiatry site and with randomly varying block sizes within the strata, between January 2015 and May 2017. Participants’ eligibility criteria have been described elsewhere [1,3]. Follow-up data collection occurred at 6- and 12-months post-randomisation by blinded research workers. Statisticians were also blinded to treatment group prior to the main outcome analysis but participants, carers, clinicians the Trial Manager and Chief Investigator were not blinded to intervention allocation.

As reported elsewhere [1], [2], [3] SMC was provided by neurologists / epilepsy specialists and liaison psychiatrists / neuropsychiatrists who had a role in the delivery of the patient's diagnosis and ongoing management. The SMC doctors gave patients information booklets (downloadable from http://www.codestrial.org/information-booklets/4579871164). They were provided with written guidelines to assist them in their explanation of the diagnosis and subsequent management. While the neurologists’ provision of SMC began with the DS diagnosis delivery, their main role in subsequent appointments was to review the patient, supervise the withdrawal of anti-seizure medications if appropriate, manage any comorbid physical problems and provide psychopharmacological interventions for depression / anxiety before the patient was assessed by a liaison psychiatrist / neuropsychiatrist. Psychiatrists began their delivery of SMC by undertaking a clinical psychiatric assessment around three months after the patients had received their diagnosis from their neurologist. They then carried out a general review of their patients, provided support and considered the possibility of psychopharmacological interventions for patients’ comorbidities. They were asked not to undertake any CBT-based interventions with trial patients. The option of referral for crisis management was available to neurologists and psychiatrists. We suggested that there should be at least two SMC sessions provided by neurologists and three to four SMC sessions from psychiatrists. As indicated above, neurologists / epilepsy specialists were based across 27 services and psychiatrists were employed in 17 services in England, Scotland and Wales.

Our model of DS-specific CBT was based broadly on the approach used in our pilot study [4]. It has been described elsewhere [[1], [2], [3],^19^,^20^]. It is based largely on models of fear-avoidance [21,22]. The model conceptualises DS as states of altered awareness and responsiveness that initially occur when the person is in a state of heightened arousal [23]. DS then give rise to behavioural and emotional avoidance through fear of having further seizures, leading to a more restricted lifestyle. The intervention was designed to include techniques to promote the reduction of DS and of behavioural and emotional avoidance, with the aim of facilitating patients’ re-engagement in everyday activities and healthy relationships. The treatment was structured to be delivered in 12 sessions over a four-to-five-month period with a later booster session at nine months post-randomisation. The intervention was manualised with a suggested structure and content for each session but allowed sufficient flexibility to be formulation-based and tailored to the individual. Therapy was delivered by one of 39 CBT therapists who in addition to previous CBT training had received study-specific training and who were allocated to study supervision groups. Therapy fidelity was evaluated and found to be acceptable [2,3]. Further details about study design, blinding, randomisation, adverse events, data collection and management are provided elsewhere [1–3]. The trial was registered as ISRCTN05681227 and ClinicalTrials.gov NCT02325544.

### 2.2 Outcome measures

For this secondary analysis we evaluated the treatment effects at 6 months post-randomisation, using an intention-to-treat analysis. As previously noted [2], [3], one patient randomised to SMC-alone received our DS-specific CBT in error.

From a theoretical and clinical perspective, we expected that our secondary analysis of outcomes would show an improvement around the end of CBT. Although limited in the number of measures we could include, our selection of outcome measures, as in the main CODES trial, focused on those we felt would change as a result of the targeted intervention, as previously described elsewhere [1–3,19,20] and which addressed DS occurrence, behavioural and emotional avoidance, emotional distress, DS-related cognitions / beliefs, trauma and social factors. Indeed, DS are a heterogeneous, multifaceted disorder which may confer disability in a range of ways, not only through seizure frequency but also their association with mental and physical disorders or social effects [24]. Thus, our outcome measures included measures of seizure occurrence, impact of seizures, distress and global improvement.

We included 14 measures all of which had been assessed at 12 months in the primary trial analysis [2].

Previous primary outcome measure:

(1) Monthly DS frequency (measured over the previous four weeks). The construction of this measure, from seizure diaries, or where not available, a single self-report measure, has been described previously [2,3].

Previous secondary outcome measures:

Outcomes related to seizure experience:

(1) Patients’ self-ratings of DS severity [6].
(2) Patients’ self-ratings of seizure ‘bothersomeness’ [6].
(3) Whether >50% reduction in DS frequency was achieved compared to baseline.

Outcomes related to health-related quality of life (HRQoL):

(1) Physical Component Summary (PCS) score from the SF-12v2 [11].
(2) Mental Component Summary (MCS) score from the SF-12v2 [11].
(3) Self-reported health today from the EQ-5D-5L visual analogue scale (VAS) [10].

Outcomes related to psychosocial functioning and impact of DS on everyday functioning:

(1) Work and Social Adjustment Scale (WSAS) [7].

Outcomes related to psychological symptoms, distress and somatic symptom burden:

(1) Anxiety scores on the Generalised Anxiety Disorder Assessment 7-item (GAD-7) [25].
(2) Depression scores on the Patient Health Questionnaire 9-item (PHQ-9) [26].
(3) General distress on the Clinical Outcomes in Routine Evaluation – 10 (CORE-10) [9] scale.
(4) Somatic symptoms: Modified PHQ-15 incorporating all 30 items [8].

Outcomes related to global impression of improvement and satisfaction with treatment:

(1) Patient self-reported change derived from the Clinical Global Impression of Improvement (CGI) rating [27].
(2) Patient-reported satisfaction with treatment using a seven-point scale.

Analyses of between group differences at 6 months post-randomisation could not include the following variables that were previously evaluated at 12 months post-randomisation [2] as they were not collected at the 6-month post-randomisation timepoint: longest number of consecutive days of seizure freedom in the last six months; seizure freedom in the last three months of the study and doctor-rated global impression of change (CGI).

### 2.3 Statistical analysis

The study was powered for the primary outcome as defined for the main trial to detect a difference in monthly seizure frequency in the CBT+SMC arm compared to the SMC-alone arm, represented by an incidence rate ratio (IRR) of around 0.66. The previously-defined primary and 16 secondary outcomes for the RCT were formally tested at 12 months post-randomisation. However, the secondary outcomes were not powered to show a difference between treatment arms. Therefore no adjustment for multiple comparisons was made. For consistency we used this same approach for the exploratory analysis of the 6-month outcomes.

The statistical approach used in this paper matched how we evaluated differences in treatment effects at 12 months. This was described in detail in our original trial publications [2,3]. The only difference was that the 6-month outcome featured as the dependant variable, rather than the 12-month outcome. Outcome variables were assessed in the intention-to-treat population with multiple imputation (MI) used to facilitate the inclusion of all randomised participants in formal analyses. The use of MI was necessary as post-randomisation variables contained missing values and treatment compliance within the CBT+SMC arm was found to be predictive of missingness at 12-month follow-up. MI provides valid inferences under a missing at random assumption if observed variables are included in the imputation step to allow prediction of missingness. Multivariate imputation by chained equations (MICE) requires the specification of an imputation model and an analysis model. The imputation models employed in this study were the same as those described previously [2,3], i.e., the following baseline predictors of missing outcome were included: longest period of seizure in the previous 6 months, number of somatic symptoms measured on the Modified PHQ-15, relationship status, having a carer and previously having sought help for a mental health problem. Other variables included were: trial arm, baseline measure of outcome, and trial centre; and all variables in the imputation step were included in the analysis models, as described previously [2,3]. Over-dispersed count variables such as seizure frequency were analysed using a negative binomial model with CBT effects expressed as IRRs. Continuous and discrete outcome variables, such as seizure severity, were analysed using linear regression with CBT effects expressed by mean differences. Finally, logistic regression modelling was used for binary variables such as achieving >50% reduction in DS frequency with CBT effects expressed by odds ratios (ORs).

All statistical analysis was performed using Stata v15 (StataCorp, Texas).

## 4 Results

**Fig. 1:**
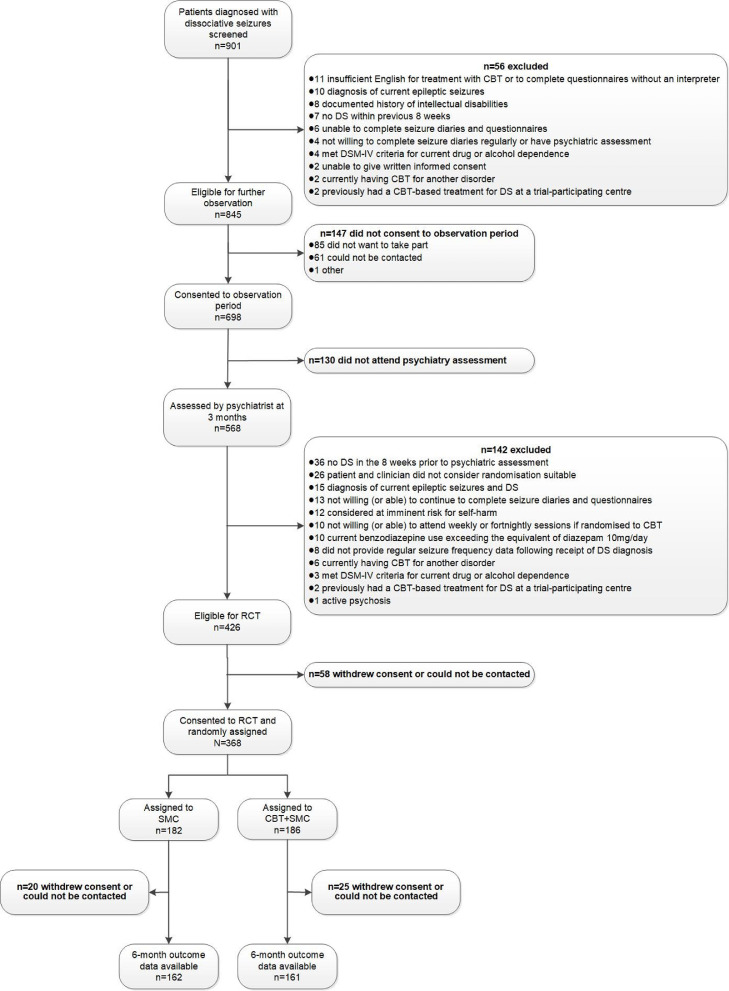
Study flowchart showing initial recruitment into the study observation period and then into the randomised controlled trial, indicating the number of participants contributing to the 6-month follow-up data evaluated in this secondary analysis. This figure is adapted from Goldstein et al. [2]. This is an Open Access article distributed in accordance with the terms of the Creative Commons Attribution (CC BY 4.0) license, which permits others to distribute, remix, adapt and build upon this work, for commercial use, provided the original work is properly cited. See: http://creativecommons.org/licenses/by/4.0/. The current figure includes formatting changes from the original figure and displays participant numbers for the 6-month data collection point rather than at the final 12-month post-randomisation data point.

While a CONSORT flow-chart leading to the 12-month follow-up in the main trial can be found in our main trial reports [2,3], Fig. 1 shows the number of participants who finally contributed to the 6-month outcomes which are considered in this secondary analysis. As described elsewhere [2,3,28] the baseline characteristics of the 368 people randomised to the RCT indicated that 266 (72%) were women. The mean age of the sample was 37.5 years (SD 14.3) and 330 (90%) were white. One hundred and ninety-five (53%) were married or living with a partner as opposed to being single, separated or widowed. Of 365 providing information about employment status, only 123 (34%) were currently employed or in education. The majority who were of working age and who were unemployed (165/233 (71%)) were in receipt of state disability benefits as were 18/110 (16%) of those who were of working age and employed. Just over half of the sample (195 (53%)) had received their diagnosis based on video-electroencephalography. The median age at onset of DS was 29 (IQR 19, 42; mode 19 years) and the median duration of their disorder was 3 years (IQR 1, 8). In addition, 236/366 (64%) patients were judged by their clinician to have predominantly hyperkinetic as opposed to predominantly hypokinetic seizures. Just over a quarter of patients (101; 27%) self-reported a previous diagnosis of epilepsy. Nearly two-thirds (241; 66%) reported previously having sought help for a mental health problem and 261/365 (72%) reported having comorbid medical conditions. When screened on the Mini-International Neuropsychiatric Interview (M.I.N.I.) [29] 255 (69%) were found to have at least one current DSM-IV diagnosis. These background demographic details are shown by treatment group as well as overall in Supplementary Table 1. Over the entire 12-month trial period the CBT+SMC group attended a median of 3 SMC sessions (IQR 2, 5; range 0–19) and the SMC-alone group attended a median of 4 SMC sessions (IQR 2, 5; range 0–12) [3].

**Fig. 2:**
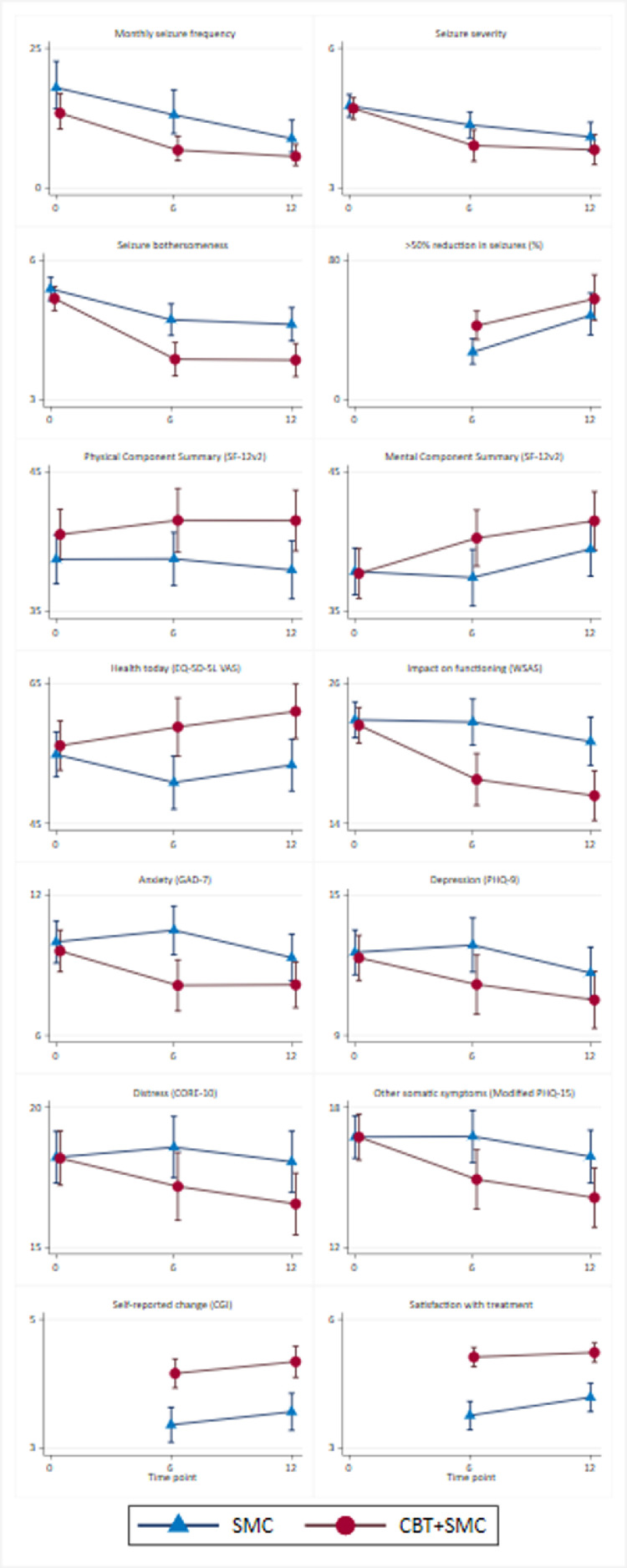
Data plots of all 14 outcomes at baseline and 6- and 12-months post-randomisation for the CBT+SMC and SMC-alone groups. Legend: Data plots depict mean scores on the relevant scales with 95% confidence intervals (as in Table 1), except for monthly seizure frequency, which is depicted by geometric means. CBT= cognitive behavioural therapy. SMC= standardised medical care. SF-12v2: Short Form 12-item (version 2) Health Survey; EQ-5D-5L: EuroQol 5-dimension 5-level; VAS: visual analogue scale; WSAS: Work and Social Adjustment Scale; GAD-7: Generalised Anxiety Disorder Assessment 7-item; PHQ-9: Patient Health Questionnaire 9-item; CORE-10: Clinical Outcomes in Routine Evaluation 10-item; PHQ-15: Patient Health Questionnaire 15-item; CGI: clinical global impression of improvement. These data plots are adapted from Goldstein et al. [2]. This is an Open Access article distributed in accordance with the terms of the Creative Commons Attribution (CC BY 4.0) license, which permits others to distribute, remix, adapt and build upon this work, for commercial use, provided the original work is properly cited. See: http://creativecommons.org/licenses/by/4.0/. The figure includes formatting changes from the original figures.

Table 1 summarises baseline and 6-month data for all outcome variables evaluated here and Fig. 2 summarises the baseline, 6- and 12-month data for the same outcomes. The results of the formal trial arm comparisons are shown in Table 2. This shows that, including monthly seizure frequency, all but one measure (the PCS score from the SF-12v2) significantly favoured CBT+SMC at the unadjusted 5% significance level. Most notably, monthly seizure frequency, seizure severity, the proportion showing >50% reduction in seizure frequency from baseline, anxiety (GAD-7), depression (PHQ-9), and the MCS score from the SF-12v2 differed between groups at 6 months in favour of CBT+SMC. In no instances did the SMC-alone group show more favourable outcomes than the CBT+SMC group.

**Table 1:** Descriptive summaries of outcome measures at baseline (pre-randomisation) and 6 months follow-up.

		Baseline			6-months		
		SMC N=182	CBT+SMC N=186	Overall N=368	SMC N=182	CBT+SMC N=186	Overall N=368
Monthly seizure frequency in last 4 weeks	median (IQR) [range]	19 (5, 49) [0, 649] *n* = 182	12.5 (4, 41) [0, 535] *n* = 186	15 (4, 47) [0, 649] *n* = 368	18 (3, 48) [0, 640] *n* = 162	6 (0, 24) [0, 849] *n* = 161	9 (1, 38) [0, 849] *n* = 323
Seizure severity 1=very mild, 7=very severe	mean (SD) [range]	4.8 (1.6) [1,7] *n* = 179	4.7 (1.6) [1,7] *n* = 182	4.7 (1.6) [1,7] *n* = 361	4.4 (1.6) [1,7] *n* = 135	3.9 (1.9) [1,7] *n* = 125	4.1 (1.8) [1,7] *n* = 260
Seizure bothersomeness 1=no bother at all, 7=very bothersome	mean (SD) [range]	5.4 (1.7) [1,7] *n* = 180	5.2 (1.7) [1,7] *n* = 182	5.3 (1.7) [1,7] *n* = 362	4.7 (2.0) [1,7] *n* = 143	3.9 (2.1) [1,7] *n* = 134	4.3 (2.1) [1,7] *n* = 277
>50% reduction in monthly seizure							
frequency relative to baseline		-	-	-	*n* = 157	*n* = 153	*n* = 310
	Yes	-	-	-	43 (27.4)	65 (42.5)	108 (34.8)
	No	-	-	-	114 (72.6)	88 (57.5)	202 (65.2)
Physical Component Summary Score (SF-12v2) 0=worst health, 100=best health	mean (SD) [range]	38.8 (11.9) [13.9, 65.6] *n* = 181	40.5 (12.4) [13.4, 65.9] *n* = 185	39.7 (12.2) [13.4, 65.9] *n* = 366	38.8 (11.4) [13.1, 59.5] *n* = 142	41.5 (13.4) [15.9, 66.7] *n* = 134	40.1 (12.4) [13.1, 66.7] *n* = 276
Mental Component Summary Score (SF-12v2) 0=worst health, 100=best health	mean (SD) [range]	37.9 (11.4) [16.9, 68.1] *n* = 181	37.7 (12.2) [13.4, 67.6] *n* = 185	37.8 (11.8) [13.4, 68.1] *n* = 366	37.5 (12.1) [10.5, 63.0] *n* = 142	40.3 (11.7) [17.4, 67.5] *n* = 134	38.8 (12.0) [10.5, 67.5] *n* = 276
Health today (EQ-5D-5L VAS) 0=worst health, 100=best health	mean (SD) [range]	54.9 (21.9) [10, 100] *n* = 181	56.2 (24.1) [1, 100] *n* = 182	55.5 (23.0) [1, 100] *n* = 363	50.9 (23.1) [0, 100] *n* = 143	58.8 (24.4) [0, 100] *n* = 135	54.7 (24.0) [0, 100] *n* = 278
Impact of DS on functioning (WSAS) (range 0–40; items: 0=not at all, 8=very severe)	mean (SD) [range]	22.9 (10.5) [0, 40] *n* = 181	22.5 (10.5) [0, 40] *n* = 185	22.7 (10.5) [0, 40] *n* = 366	22.7 (11.9) [0, 40] *n* = 143	17.8 (13.1) [0, 40] *n* = 135	20.3 (12.7) [0, 40] *n* = 278
Anxiety (GAD-7) (range 0–21; items: 0=not at all, 3=nearly every day)	mean (SD) [range]	10 (6.2) [0, 21] *n* = 182	9.6 (6.2) [0, 21] *n* = 186	9.8 (6.2) [0, 21] *n* = 368	10.5 (6.3) [0, 21] *n* = 143	8.1 (6.5) [0, 21] *n* = 135	9.4 (6.5) [0, 21] *n* = 278
Depression (PHQ-9) (range 0–27; items: 0=not at all, 3=nearly every day)	mean (SD) [range]	12.6 (6.5) [0, 26] *n* = 181	12.3 (6.7) [0, 27] *n* = 186	12.4 (6.6) [0, 27] *n* = 367	12.9 (7) [0, 27] *n* = 142	11.2 (7.4) [0, 27] *n* = 135	12.1 (7.2) [0, 27] *n* = 277
Distress (CORE-10) (range 0–40; items: 0=not at all, 4=all of the time)	mean (SD) [range]	18.2 (6.3) [4,34] *n* = 182	18.2 (6.7) [4,32] *n* = 186	18.2 (6.5) [4,34] *n* = 368	18.6 (6.6) [2.2, 34] *n* = 142	17.2 (7.1) [0, 39] *n* = 135	17.9 (6.9) [0, 39] *n* = 277
Other somatic symptoms (Modified PHQ-15) (range 0–30)	mean (SD) [range]	16.7 (6.2) [2,30] *n* = 181	16.7 (6.8) [2,30] *n* = 183	16.7 (6.5) [2,30] *n* = 364	16.8 (6.7) [0, 29] *n* = 140	14.9 (7.4) [0, 28] *n* = 135	15.9 (7.1) [0, 29] *n* = 275
Self-reported change (CGI) 0=very much worse, 6=very much better	mean (SD) [range]	-	-	-	3.4 (1.6) [0, 6] *n* = 140	4.2 (1.3) [0, 6] *n* = 135	3.8 (1.5) [0, 6] *n* = 275
Satisfaction with treatment (patient-reported) 0=very dissatisfied, 6=very satisfied	mean (SD) [range]	-	-	-	3.8 (2.0) [0, 6] *n* = 140	5.1 (1.3) [0, 6] *n* = 135	4.4 (1.8) [0, 6] *n* = 275

N: total; SMC: standardised medical care; CBT: cognitive behavioural therapy; IQR: inter-quartile range; SD: standard deviation; SF-12v2: Short Form 12-item (version 2) Health Survey; EQ-5D-5L: EuroQol 5-dimension 5-level; VAS: visual analogue scale; WSAS: Work and Social Adjustment Scale; GAD-7: Generalised Anxiety Disorder Assessment 7-item; PHQ-9: Patient Health Questionnaire 9-item; CORE-10: Clinical Outcomes in Routine Evaluation 10-item; PHQ-15: Patient Health Questionnaire 15-item; CGI: clinical global impression of improvement. Adapted from Goldstein et al [2]. This is an Open Access article distributed in accordance with the terms of the Creative Commons Attribution (CC BY 4.0) license, which permits others to distribute, remix, adapt and build upon this work, for commercial use, provided the original work is properly cited. See: http://creativecommons.org/licenses/by/4.0/. The table includes formatting changes from the original tables.

**Table 2:** Between group differences at 6 months post-randomisation.

	6 months post-randomisation		
	Estimated trial arm difference (original scale)	95% CI	P-value
Monthly seizure frequency	IRR=0.72	(0.55, 0.93)	0.013
Seizure severity	-0.37	(-0.74, -0.01)	0.045
Seizure bothersomeness	-0.66	(-1.06, -0.26)	0.001
>50% reduction in monthly seizure frequency relative to baseline	OR=2.17	(1.34, 3.52)	0.002
Physical Component Summary score (SF-12v2)	1.07	(-0.86, 3.00)	0.278
Mental Component Summary score (SF-12v2)	3.20	(0.85, 5.55)	0.008
Health today (EQ-5D-5L VAS)	6.83	(1.93, 11.73)	0.006
Impact on functioning (WSAS)	-4.74	(-6.80, -2.68)	<0.001
Anxiety (GAD-7)	-2.18	(-3.36, -1.00)	<0.001
Depression (PHQ-9)	-1.74	(-2.92, -0.56)	0.004
Distress (CORE-10)	-1.63	(-2.95, -0.32)	0.015
Other somatic symptoms (Modified PHQ-15)	-2.04	(-3.29, -0.80)	0.001
Self-reported change (CGI)	0.78	(0.45, 1.12)	<0.001
Satisfaction with treatment (patient-reported)	1.13	(0.84, 1.62)	<0.001

SF-12v2: Short Form 12-item (version 2) Health Survey; EQ-5D-5L: EuroQol 5-dimension 5-level; VAS: visual analogue scale; WSAS: Work and Social Adjustment Scale; GAD-7: Generalised Anxiety Disorder Assessment 7-item; PHQ-9: Patient Health Questionnaire 9-item; CORE-10: Clinical Outcomes in Routine Evaluation 10-item; PHQ-15: Patient Health Questionnaire 15-item; CGI: clinical global impression of improvement

**Fig. 3:**
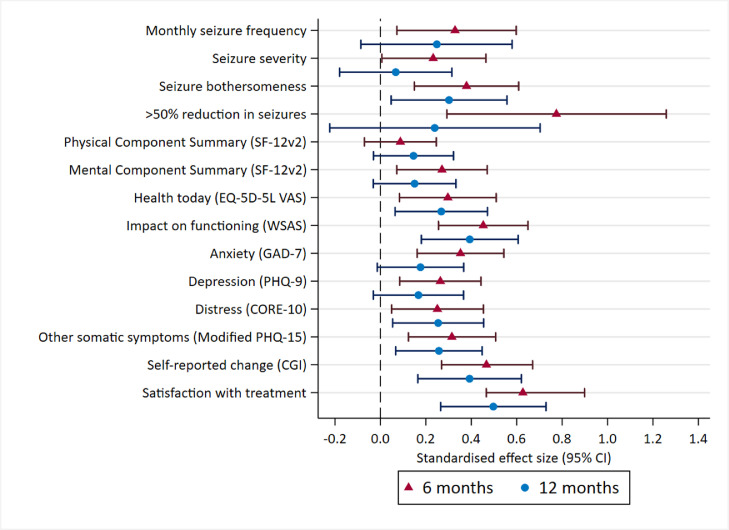
Forest plot showing standardised effect sizes for all outcome measures assessed both at 6- and 12-months follow-up Legend: Standardised effect sizes were calculated using the following methods: (a) for continuous outcomes, the estimated difference between arms was divided by the standard deviation of the baseline measure; (b) if there was no baseline measure, the difference was divided by the pooled standard deviation of the outcome; and (c) for count or binary outcomes, the estimated IRR or OR was log-transformed. This figure is adapted from Goldstein et al. [2]. This is an Open Access article distributed in accordance with the terms of the Creative Commons Attribution (CC BY 4.0) license, which permits others to distribute, remix, adapt and build upon this work, for commercial use, provided the original work is properly cited. See: http://creativecommons.org/licenses/by/4.0/. The figure includes additions and formatting changes to the original figure. .

Fig. 3 displays the standardised group differences (effect sizes) for all outcome measures at 6 and 12 months (standardised group differences for the continuous outcomes were previously reported at 12 months [2,3]). The average effect size of the continuous outcomes was 0.33 at 6 months in comparison to 0.26 at 12 months. The estimated IRR quantifying the seizure reduction was IRR = 0.72 (95%CI from 0.55 to 0.93) at 6 months, in comparison to IRR = 0.78 at 12 months [2], [3]. The CBT effect in terms of >50% reduction in monthly seizure frequency relative to baseline at 6 months was OR = 2.17 (95%CI from 1.34 to 3.52) compared to OR = 1.27 at 12 months [2], [3].

## 5 Discussion

We investigated differences between groups in our RCT comparing DS-specific CBT + SMC with SMC alone. Our exploratory secondary analyses found that all but one of the outcomes were significantly different in favour of CBT+SMC at the unadjusted 5% level, with large-to-moderate effect sizes at the 6-month post-randomisation point. This included monthly DS frequency, the predefined primary outcome measure at the 12-month follow-up point. The findings are consistent with the impression gained from the unadjusted raw data and data plots over time reported in our previous paper [2,3] and in Fig. 2 here. Fewer significant differences were found at 12 months post-randomisation but this paper focuses on the 6-month follow-up, which occurred at a time broadly corresponding to the end of active regular treatment sessions.

The use of independent analyses at 6- and 12-months post-randomisation makes a formal comparison between two timepoints difficult. The data and effect sizes at 6 and 12 months (Figs. 2 and 3) give the impression of clear between-group differences at 6 months across almost all outcome measures. These were slightly weaker in most instances at 12 months. The clearest exception to this is the impact of seizures reflected by WSAS scores, where the CBT effect was found to be highly significant at both 6 and 12 months. This is likely to reflect the strong emphasis on reducing avoidance behaviour in the DS-specific CBT intervention. Self-rated improvement was also clearly significant at both timepoints as was satisfaction with treatment. These three findings (WSAS scores, self-rated improvement and satisfaction with treatment) seem, therefore, to indicate better maintained between-group treatment effects. There was a clear CBT+SMC group advantage at both timepoints in terms of self-ratings of seizure ‘bothersomeness’, suggesting that the CBT intervention appeared to have led patients to better tolerate ongoing seizures. There was also evidence of maintained benefits in terms of measures of distress (CORE-10), somatic symptoms (Modified PHQ-15) and ratings of health today (EQ-5D-5L VAS). The effect sizes were not as large at 12 months. Nevertheless, the fact that differences between groups were maintained in these outcomes suggests that the treatment effects were long lasting.

The current finding that DS frequency was significantly lower in the CBT+SMC group than in the SMC-alone group at the 6-month post-randomisation follow-up, supports the findings of our previous pilot study [4] where DS frequency was significantly lower at treatment end following CBT. Indeed, the overall pattern of DS frequency over time is very similar to that seen in the earlier study. In that study we may have been underpowered to detect between-group differences in secondary outcomes, which in the CODES trial we were better able to do.

We have commented previously [2,3] that our SMC intervention contained many active treatment ingredients. Although we did not formally monitor psychopharmacological interventions by the SMC clinicians, providing a coherent explanation for seizure maintenance, support, review and, where appropriate, psychotropic medication prescription resulted in some benefit. As part of SMC, patients received what can be conceptualised as standardised, but also specialist, medical care that involved input from neurologists and psychiatrists who had been provided with study materials to guide their diagnosis delivery and explanation of the disorder. In addition, clinicians were permitted to describe (but were asked not to not practise with trial patients) seizure distraction techniques to avert seizures. They were also able to direct participants to self-help websites which contained information on seizure control techniques.

Our raw data tables and plots (Fig. 2 and Goldstein et al. [2,3]) lead us to speculate that, rather than the change in significance in the between-group differences being attributable to diminished benefit in the CBT+SMC group, the weakening of between-groups significance could be explained by improvements in the SMC-alone group at 12 months. Since SMC was not a treatment-as-usual comparison arm, it is possible that our specialist intervention, with sessions that were often spread across the 12-month post-randomisation period and content that was potentially therapeutic, led to gradual improvement in the SMC-alone group, reducing between-group differences. Although the CBT+SMC group advantage in terms of DS frequency reduction at the end of active treatment (6 months) may relate to the inclusion of DS control techniques as part of the CBT intervention, the SMC-alone group may have been experiencing beneficial exposure to distraction techniques and online materials [30], leading to improvement.

CBT+SMC participants seemed to show gains in relation to seizure impact as assessed by the WSAS which asks specifically about work. However, whether these gains were accompanied by improved actual work status is unknown. Again, while we cannot know which components of the CBT approach were selectively effective, our DS-specific CBT did address avoidance behaviour, and this may correlate with improvements seen on the WSAS.

Our investigation here of the 6-month differences between trial arms raises the possibility of more rapid change / improvement in the CBT+SMC group versus SMC alone. We recognise that better outcomes at 6 months may be valuable to many patients, even if some between-group differences are smaller at 12 months. However, since we did not match groups for therapist contact, we cannot rule out the possibility of an increased contact effect rather than a true CBT-effect in terms of seizure frequency in the CBT+SMC vs SMC-alone groups. For informed roll-out of the intervention, it is important to understand which baseline characteristics interact with the CBT treatment effect to bring about greater improvement. We will, therefore, be exploring moderators of treatment effects in a future paper.

The lack of adjustment for multiple comparisons may be considered to be a limitation of this study but our approach was consistent with the analysis of the 12-month outcome data [2,3,31]. In addition to adopting a consistent approach, most methods of adjustment for multiple comparisons are overly-conservative, and are more relevant when trying to claim results are fully powered, which as we noted earlier they were not. We were more interested in the clinical significance of the 6-month results in the context of the overall study, rather than the p-values per se.

## 6 Conclusion

Overall, our analyses suggest that while at 12 months post-randomisation our predesignated primary outcome (monthly seizure frequency) was no longer significantly different between groups [2,3], despite significant benefits of CBT+SMC on other outcomes, there is evidence of more rapid benefit in the CBT+SMC group. This includes DS frequency reduction. This pattern of results can potentially be attributed to improvements made in the SMC-alone group at 12 months which were not as apparent at 6 months (Fig. 2) and demonstrates the importance of longer follow-up outcome evaluations in treatment trials [12]. It suggests caution should be exercised when reviewing positive trial outcomes with only short follow-up periods.

## Ethical publication statement

We confirm that we have read the Journal's position on issues involved in ethical publication and affirm that this report is consistent with those guidelines. Ethical approval was granted by the London-Camberwell St Giles NRES Committee (reference number 13/LO/1595).

## Declaration of Competing Interest

AC reports being a paid editor of the Journal of Neurology, Neurosurgery and Psychiatry, and is the director of a research programme on functional neurological disorders; he gives independent testimony in Court on a range of neuropsychiatric topics (50% pursuer, 50% defender). SL is a paid editor of the Journal of Child Psychology and Psychiatry. MR is the paid Editor-in-Chief of Seizure - European Journal of Epilepsy and receives authorship fees from Oxford University Press in relation to a number of books about dissociative seizures. JS reports independent expert testimony work for personal injury and medical negligence claims, royalties from UpToDate for articles on the functional neurological disorder and runs a free non-profit self-help website, www.neurosymptoms.org. The remaining authors have no conflicts of interest to declare.

## Appendix Supplementary materials

Image, application 1
