# Supplementary material for: Six-month outcomes of the CODES randomised controlled trial of cognitive behavioural therapy for dissociative seizures: A secondary analysis
Source: Seizure. 2022 Mar;96:128–36. doi: 10.1016/j.seizure.2022.01.016 (PMC8970049; doi:10.1016/j.seizure.2022.01.016)
Supplement: Supplementary file 1 [file mmc1.docx]

**Supplementary Table 1 Demographic and clinical characteristics of participants in each treatment arm and overall pre-randomisation.**

| **Characteristic** | **SMC (N=182)** | **CBT+SMC (N=186)** | **Overall (N=368)** |
| --- | --- | --- | --- |
| Gender n (%)  Male  Female | 56 (30.8)  126 (69.2) | 46 (24.7)  140 (75.3) | 102 (27.7)  266 (72.3) |
| Age (years)  Mean (sd) | 37.7 (14.5) | 37.3 (14.2) | 37.5 (14.3) |
| Ethnicity n (%)  White  Other | 163 (89.6)  19 (10.4) | 167 (89.8)  19 (10.2) | 330 (89.7)  38 (10.3) |
| Relationship status n (%)  Married / living with partner  Single / separated / widowed | 97 (53.3)  85 (46.7) | 98 (52.7)  88 (47.3) | 195 (53.0)  173 (47.0) |
| Employed / in education n (%) | (n=180)  58 (32.2) | (n=185)  65 (35.1) | (n=365)  123 (33.7) |
| In receipt of state disability benefits if of working age (<65 years) n (%)  Yes (not working)  Yes (working) | (n=115)  86 (74.8)  (n=58)  13 (22.4) | (n=118)  79 (66.9)  (n=52)  5 (9.6) | (n=233)  165 (70.8)  (n=110)  18 (16.4) |
| Diagnosed using video-EEG n (%) | 94 (51.6) | 101 (54.3) | 195 (53.0) |
| Age at onset of DS (years)  median (IQR) [range] | (n=181)  29 (19, 42) [5, 76] | (n=184)  29 (19, 41.5) [1, 67] | (n=365)  29 (19, 42) [1, 76] |
| Number of years with DS  median (IQR) [range] | (n=181)  3 (1, 8)  [0, 65] | (n=184)  3 (1, 7.5)  [0, 44] | (n=365)  3 (1, 8)  [0, 65] |
| Predominant DS semiology n (%)  Hyperkinetic  Hypokinetic | (n=181)  121 (66.9)  60 (33.1) | (n=185)  115 (62.2)  70 (37.8) | (n=366)  236 (64.4)  130 (35.6) |
| Previous diagnosis of epilepsy (patient-reported) n (%) | 52 (28.6) | 49 (26.3) | 101 (27.4) |
| Previously sought medical help for mental health problem n (%) | 116 (63.7) | 125 (67.2) | 241 (65.5) |
| Currently suffering from any other medical problem n (%) | (n=181)  131 (72.4) | (n=184)  130 (70.7) | (n=365)  261 (71.5) |
| At least 1 current DSM-IV diagnoses from the M.I.N.I. n (%) | 125 (68.7) | 130 (69.9) | 255 (69.3) |

DS= dissociative seizures N: total sample size; SMC: standardised medical care; CBT: cognitive behavioural therapy; SD: standard deviation; IQR: inter-quartile range; EEG: electroencephalogram; DSM-IV: Diagnostic and Statistical Manual of Mental Disorders, 4th Edition; M.I.N.I.: Mini - International Neuropsychiatric Interview. All data are complete unless otherwise specified.

This table is adapted from in Goldstein et al (2020)*. This is an Open Access article distributed in accordance with the terms of the Creative Commons Attribution (CC BY 4.0) license, which permits others to distribute, remix, adapt and build upon this work, for commercial use, provided the original work is properly cited. See: http://creativecommons.org/licenses/by/4.0/. The Table includes minor additions and formatting changes to the original table, which had included additional information.

*Goldstein LH, Robinson EJ, Mellers JDC, Stone J, Carson A, Reuber M, et al. Cognitive behavioural therapy for adults with dissociative seizures (CODES): a pragmatic, multicentre, randomised controlled trial. The Lancet Psychiatry 2020;7: 491-505.
